# Draft Genome Sequence of *Escherichia coli* Strain M15-4, a Typical Enteropathogenic *E. coli* Strain Isolated in Mexico

**DOI:** 10.1128/genomeA.01522-17

**Published:** 2018-02-01

**Authors:** José Antonio Magaña-Lizárraga, Yesmi Patricia Ahumada-Santos, Jesús Ricardo Parra-Unda, Magdalena de J. Uribe-Beltrán, Bruno Gómez-Gil, María Elena Báez-Flores

**Affiliations:** a Unidad de Investigaciones en Salud Pública Dra. Kaethe Willms, Facultad de Ciencias Químico Biológicas, Universidad Autónoma de Sinaloa, Culiacán, Sinaloa, México; b Centro de Investigación en Alimentación y Desarrollo, A. C. Mazatlán, Sinaloa, México

## Abstract

We present here the first draft genome sequence of a typical enteropathogenic *Escherichia coli* serotype O55:H51 strain, M15-4, isolated from a 2-month-old infant girl with acute diarrhea. The study of this Mexican isolate will provide insights to the virulence and drug resistance traits involved in its pathogenic potential.

## GENOME ANNOUNCEMENT

According to the World Health Organization, diarrheal disease is the second leading cause of death in children under 5 years old and kills around 525,000 children every year (http://www.who.int/mediacentre/factsheets/fs330/en). Typical enteropathogenic *Escherichia coli* (tEPEC) strains, which belong to one of the diarrheagenic *E. coli* pathotypes, remain a significant public health concern in low-income countries owing to their high rates of hospitalization and mortality in infants with moderate to severe diarrhea, particularly in children under the age of 12 months (1). Here, we report the draft genome sequence of tEPEC strain M15-4, isolated from a 2-month-old infant girl with acute diarrhea. Identification of strain M15-4 was completed on the Vitek system and classified as a tEPEC strain based on the positive PCR amplification of the *eaeA* and *bfpA* determinants (2).

The genome sequencing of *E. coli* M15-4 was performed using Ion Torrent PGM technology (Life Technologies, Inc., Carlsbad, CA, USA) with 200-bp chemistry on a 316 chip. The sequencing generated 369,712 reads with a mean length of 204 bp. *De novo* assembly was performed with SPAdes version 3.11 (3), resulting in 192 contigs with a maximum length of 283,282 bp, an *N*_50_ of 71,091 bp, a G+C content of 50.48%, and a predicted genome size of 5,082,644 bp. Gene prediction and genome annotation were performed with the NCBI Prokaryotic Genome Annotation Pipeline (PGAP) version 4.2 (4) and the Rapid Annotations using Subsystems Technology (RAST) server (5). SeroTypeFinder version 1.1, VirulenceFinder version 1.5, and ResFinder version 3.0 were used to determine serotype, virulence factors, and antibiotic resistance genes in this isolate, respectively (6–8). PGAP analysis revealed 5,513 genes, 5,420 protein-coding sequences, and 93 RNA genes (16 rRNAs, 71 tRNAs, and 6 noncoding RNAs). According to the RAST nearest-neighbor analysis, the closest genome is that of *E. coli* AA86. Furthermore, 101 features corresponded to resistance to antibiotics (beta-lactams, fluoroquinolones, and aminoglycosides), and toxic substances (copper, cobalt, zinc, cadmium, and mercury) were found. The isolate displayed an O55:H51 serotype, and the tEPEC pathotype was confirmed by the presence of *eae*, *tir*, *perA* (EPEC adherence factor), *bfpA*, and several pathogenicity-related genes in its genome. Interestingly, we found a sequence corresponding to *capU*, a gene belonging to a plasmid-associated cap cluster, which is involved in the biofilm formation of the enteroaggregative *E. coli* (EAEC) pathotype (9). Moreover, genes conferring aminoglycoside, beta-lactam, sulfonamide, tetracycline, and trimethoprim resistance [*aadA1*, *bla*_TEM-1B_, *sul1*, *tet*(A), and *dfrA15*, respectively] were identified. To the best of our knowledge, this is the first reported sequence of an *E. coli* serotype O55:H51 strain. Likewise, this is the first time that the *capU* gene has been identified in a tEPEC strain.

### Accession number(s).

This whole-genome shotgun project has been deposited at DDBJ/ENA/GenBank under the accession number PDCE00000000. The version described in this paper is the first version, PDCE01000000.

## References

[1] Kotloff KL, Nataro JP, Blackwelder WC, Nasrin D, Farag TH, Panchalingam S, Wu Y, Sow SO, Sur D, Breiman RF, Faruque ASG, Zaidi AK, Saha D, Alonso PL, Tamboura B, Sanogo D, Onwuchekwa U, Manna B, Ramamurthy T, Kanungo S, Ochieng JB, Omore R, Oundo JO, Hossain A, Das SK, Ahmed S, Qureshi S, Quadri F, Adegbola RA, Antonio M, Hossain MJ, Akinsola A, Mandomando I, Nhampossa T, Acácio S, Biswas K, O’Reilly CE, Mintz ED, Berkeley LY, Muhsen K, Sommerfelt H, Robins-Browne RM, Levine MM 2013 Burden and aetiology of diarrhoeal disease in infants and young children in developing countries (the Global Enteric Multicenter Study, GEMS): a prospective, case-control study. Lancet 382:209–222. doi:10.1016/S0140-6736(13)60844-2. 23680352

[2] López-Saucedo C, Cerna JF, Villegas-Sepulveda N, Thompson R, Velazquez FR, Torres J, Tarr PI, Estrada-García T 2003 Single multiplex polymerase chain reaction to detect diverse loci associated with diarrheagenic *Escherichia coli*. Emerg Infect Dis 9:127–131. doi:10.3201/eid0901.010507. 12533296 PMC2873745

[3] Bankevich A, Nurk S, Antipov D, Gurevich AA, Dvorkin M, Kulikov AS, Lesin VM, Nikolenko SI, Pham S, Prjibelski AD, Pyshkin AV, Sirotkin AV, Vyahhi N, Tesler G, Alekseyev MA, Pevzner PA 2012 SPAdes: a new genome assembly algorithm and its applications to single-cell sequencing. J Comput Biol 19:455–477. doi:10.1089/cmb.2012.0021. 22506599 PMC3342519

[4] Tatusova T, DiCuccio M, Badretdin A, Chetvernin V, Nawrocki EP, Zaslavsky L, Lomsadze A, Pruitt KD, Borodovsky M, Ostell J 2016 NCBI prokaryotic genome annotation pipeline. Nucleic Acids Res 44:6614–6624. doi:10.1093/nar/gkw569. 27342282 PMC5001611

[5] Aziz RK, Bartels D, Best AA, DeJongh M, Disz T, Edwards RA, Formsma K, Gerdes S, Glass EM, Kubal M, Meyer F, Olsen GJ, Olson R, Osterman AL, Overbeek RA, McNeil LK, Paarmann D, Paczian T, Parrello B, Pusch GD, Reich C, Stevens R, Vassieva O, Vonstein V, Wilke A, Zagnitko O 2008 The RAST server: Rapid Annotations using Subsystems Technology. BMC Genomics 9:75. doi:10.1186/1471-2164-9-75. 18261238 PMC2265698

[6] Joensen KG, Tetzschner AMM, Iguchi A, Aarestrup FM, Scheutz F 2015 Rapid and easy *in silico* serotyping of *Escherichia coli* isolates by use of whole-genome sequencing data. J Clin Microbiol 53:2410–2426. doi:10.1128/JCM.00008-15. 25972421 PMC4508402

[7] Zankari E, Hasman H, Cosentino S, Vestergaard M, Rasmussen S, Lund O, Aarestrup FM, Larsen MV 2012 Identification of acquired antimicrobial resistance genes. J Antimicrob Chemother 67:2640–2644. doi:10.1093/jac/dks261. 22782487 PMC3468078

[8] Joensen KG, Scheutz F, Lund O, Hasman H, Kaas RS, Nielsen EM, Aarestrup FM 2014 Real-time whole-genome sequencing for routine typing, surveillance, and outbreak detection of verotoxigenic *Escherichia coli*. J Clin Microbiol 52:1501–1510. doi:10.1128/JCM.03617-13. 24574290 PMC3993690

[9] Fujiyama R, Nishi J, Imuta N, Tokuda K, Manago K, Kawano Y 2008 The *shf* gene of a *Shigella flexneri* homologue on the virulent plasmid pAA2 of enteroaggregative *Escherichia coli* 042 is required for firm biofilm formation. Curr Microbiol 56:474–480. doi:10.1007/s00284-008-9115-y. 18293034

